# Tobacco seeds expressing feedback-insensitive cystathionine gamma-synthase exhibit elevated content of methionine and altered primary metabolic profile

**DOI:** 10.1186/1471-2229-13-206

**Published:** 2013-12-07

**Authors:** Ifat Matityahu, Itamar Godo, Yael Hacham, Rachel Amir

**Affiliations:** 1 Laboratory of Plant Science, Migal Galilee Technology Center, P.O. Box 831, Kiryat Shmona 12100, Israel; 2 Tel Hai College, Upper Galilee, Israel

**Keywords:** Amino acids, Cystathionine γ-synthase, Germination, Methinonine, Metabolism, Seeds, Storage proteins

## Abstract

**Background:**

The essential sulfur-containing amino acid methionine plays a vital role in plant metabolism and human nutrition. In this study, we aimed to elucidate the regulatory role of the first committed enzyme in the methionine biosynthesis pathway, cystathionine γ-synthase (CGS), on methionine accumulation in tobacco seeds. We also studied the effect of this manipulation on the seed’s metabolism.

**Results:**

Two forms of Arabidopsis CGS (AtCGS) were expressed under the control of the seeds-specific promoter of legumin B4: feedback-sensitive *F-*AtCGS (LF seeds), and feedback-insensitive T-AtCGS (LT seeds). Unexpectedly, the soluble content of methionine was reduced significantly in both sets of transgenic seeds. Amino acids analysis and feeding experiments indicated that although the level of methionine was reduced, the flux through its synthesis had increased. As a result, the level of protein-incorporated methionine had increased significantly in LT seeds by up to 60%, but this was not observed in LF seeds, whose methionine content is tightly regulated. This increase was accompanied by a higher content of other protein-incorporated amino acids, which led to 27% protein content in the seeds although this was statistically insignificantly. In addition, the levels of reducing sugars (representing starch) were slightly but significantly reduced, while that of oil was insignificantly reduced. To assess the impact of the high expression level of T-AtCGS in seeds on other primary metabolites, metabolic profiling using GC-MS was performed. This revealed significant alterations to the primary seed metabolism manifested by a significant increase in eight annotated metabolites (mostly sugars and their oxidized derivatives), while the levels of 12 other metabolites were reduced significantly in LT compared to wild-type seeds.

**Conclusion:**

Expression of T-AtCGS leads to an increase in the level of total Met, higher contents of total amino acids, and significant changes in the levels of 20 annotated metabolites. The high level of oxidized metabolites, the two stress-associated amino acids, proline and serine, and low level of glutathione suggest oxidative stress that occurs during LT seed development. This study provides information on the metabolic consequence of increased CGS activity in seeds and how it affects the seed’s nutritional quality.

## Background

The level of methionine (Met), the essential sulfur-containing amino acid, limits the nutritional quality of crops. Met is also a fundamental metabolite in plants, since, in addition to its role as a protein constituent and its central role in the initiation of mRNA translation, it is also a precursor for the synthesis of essential metabolites through its first metabolite, *S*-adenosyl Met (SAM). Due to the nutritional and metabolic importance of Met, studies were performed to assess the factors that regulate its synthesis and accumulation in vegetative tissues (reviewed by [1-3]). However, only a limited number of studies have been performed to reveal the factors regulating its synthesis and accumulation in seeds (review by [4,5]).

**Figure 1:**
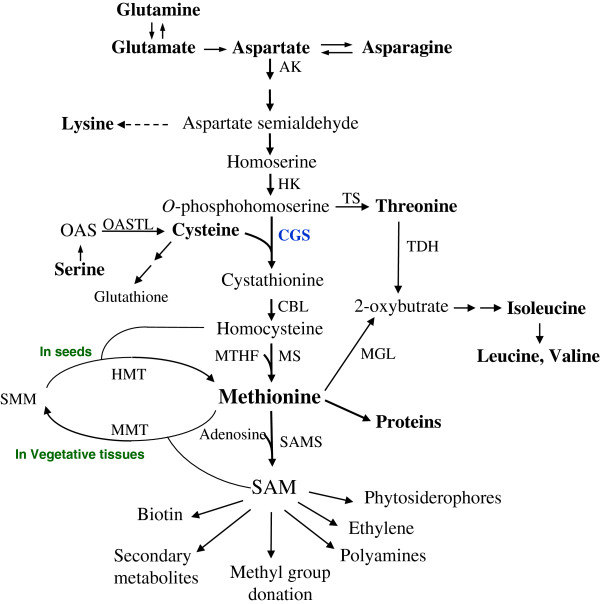
**The aspartate family of amino acids and biosynthesis pathways leading to methionine in seeds of higher plants.** Key enzymes and metabolites are specified. Abbreviations: AK, aspartate kinase; HK, homoserine kinase; TS, threonine synthase; TDH, threonine dehydratase; CGS, cystathionine γ-synthase; CBL, cystathionine β-lyase; MS, methionine synthase; OAS, *O*-acetyl serine; OASTL, *O*-acetylserine(thiol)lyase; SAM, *S*-adenosylmethionine; SAMS, SAM synthase; MGL, methionine γ-lyase; SMM, *S*-methylmethionine; MMT, methionine *S*-methyltransferase; HMT, homocysteine methyltransferase; MTHF, methyl-tetra hydrofolate.

Biochemical and genetic evidence show that Met is synthesized *de novo* in seeds through the aspartate family pathway (Figure 1) as it occurs in leaves. The first committed enzyme of the Met biosynthesis pathway, cystathionine γ-synthase (CGS), regulates the pathway by combining the carbon-amino skeleton (derived from aspartate) with the sulfur group (derived from cysteine) [3,6] (Figure 1). However, other studies performed in several plants such as Arabidopsis and wheat indicate that Met that was synthesized in vegetative tissues was converted to *S*-methyl Met (SMM) and then transported from these tissues to the developing seeds. In seeds, SMM is then converted back to Met by the activity of homocysteine *S*-methyltransferase (HMT) [7,8] (Figure 1).

While the debate about the role of SMM and aspartate pathways in Met synthesis in seeds is still ongoing, two recent studies reported that seed-specific expression of feedback-insensitive mutated forms of Arabidopsis CGS (AtCGS) in legume seeds [9,10] lead to higher levels of Met. These experiments indicate that CGS plays a major role in controlling Met synthesis in legumes seeds. However, further studies are required to reveal the roles of CGS (in the aspartate family pathway) and HMT (in the SMM pathway) in seeds of other plants.

In the current study, we aimed at revealing the role of CGS in Met synthesis in tobacco (*Nicotiana tabacum)* seeds that belong to Solanaceae. Tobacco plants were selected for this study since we had used them previously as a model plant to study the regulatory role of Arabidopsis CGS (AtCGS) in Met accumulation in tobacco vegetative tissues [11-14]. Two forms of AtCGS were overexpressed in tobacco plants: full-length AtCGS (F-AtCGS) that is feedback-sensitive to high levels of Met/SAM [15]; and truncated AtCGS (T-AtCGS) that is Met/SAM feedback-insensitive [11]. While the level of soluble Met increased significantly in plants overexpressing the F-AtCGS by about two-fold compared to wild-type (WT), those overexpressing the T-AtCGS showed a 2.5-fold increase in Met but significantly higher levels of Met that incorporated to proteins (about two-fold compared to those expressing the F-AtCGS) [11,12]. Tobacco plants were chosen for the study also since their capsule morphology enables us to apply Met or other metabolites to the developing capsules in order to assess the effect on amino acids and primary metabolites in the developing seeds [16].

In the current study, we aimed (i) to elucidate the role of CGS in Met synthesis in tobacco seeds; (ii) to study the role of the feedback inhibition of AtCGS on Met synthesis in seeds; and (iii) to study the metabolic consequences of enhanced CGS activity in these seeds.

The metabolic consequences of increased Met content have not yet been studied, and only a few studies have addressed the relationships between increased levels of other amino acids to seeds metabolism [17,18]. Developing seeds are an excellent system for studying developmentally controlled metabolic regulation because during seed development, there is a massive synthesis of fatty acids, sugars and amino acids, which are converted to their storage forms: proteins, starch and oil [19]. The results show that high expression level of T-AtCGS leads to higher total Met content and affect the level of 20 annotated metabolites. The results imply that oxidative stress might occur in the transgenic seeds expressing this form of CGS.

## Results and discussion

### The endogenous tobacco CGS is regulated by a high level of Met

**Figure 2:**
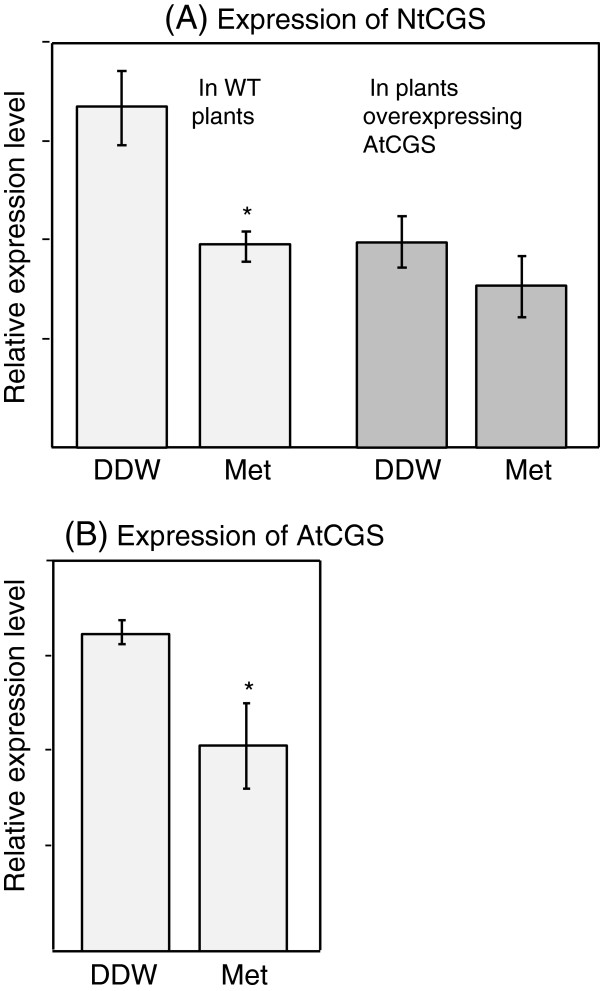
**Quantitative real-time PCR analyses of (A) NtCGS in 21-day-old seedlings of wild-type (WT) tobacco plants(left panel) and in transgenic tobacco seedlings overexpressing F-AtCGS (right panel); and of (B) AtCGS in transgenic tobacco 21-day-old seedlings overexpressing F-AtCGS.** The seedlings supplemented with distilled water (DDW) or 5 mM Met for 6 h. The values presented are the mean ± SD of five biological replicates, each with three technical replicates. Statistically significant changes (p < 0.05, using two-way ANOVA) are identified by an asterisk.

In this study, we express in tobacco seeds the feedback-sensitive form of AtCGS (F-AtCGS) and its mutated form that is insensitive to Met (T-AtCGS). To distinguish between the effects of these two forms of AtCGS, it is important to know if the endogenous tobacco CGS (NtCGS) is down-regulated by a high content of Met similarly to AtCGS [15]. To determine this, tobacco seedlings (21 days old) were fed with 5 mM Met, or with double distilled water (DDW) as a control, and the expression level of NtCGS was measured by quantitative real-time PCR (qRT-PCR). The results (Figure 2A, left panel) show that the level of NtCGS is sensitive to high levels of Met such as AtCGS and tomato CGS [20,21], but unlike potatoes [22] that belong to the Solanaceae family together with tobacco and tomato.

Most probably due to the major role of Met in plant metabolism, we expected that similar to Arabidopsis [38], NtCGS would express in all tissues of the tobacco plants. Thus, we assume that if a higher level of Met will be found in the transgenic seeds expressing the AtCGS, it will be the result of the higher activity of AtCGS and less from the endogenous NtCGS.

### Seeds expressing AtCGS do not accumulate higher levels of soluble Met

To assess whether CGS expression is a rate-limiting factor of Met synthesis in tobacco seeds, two forms of AtCGS (F-AtCGS; T-AtCGS) were expressed under the control of the seed-specific promoter, Legumin B4 [23], to produce LF and LT plants, respectively (Additional file 1: Figure S1). Seeds from 30 kanamycin-resistant plants from each line were screened by immunoblot to determine the expression level of the two forms of AtCGS (Additional file 1: Figure S2). Two plants showing the highest expression level for each line were self-pollinated to produce homozygous plants. Seeds from T_3_ lines were used for further analysis.

**Figure 3:**
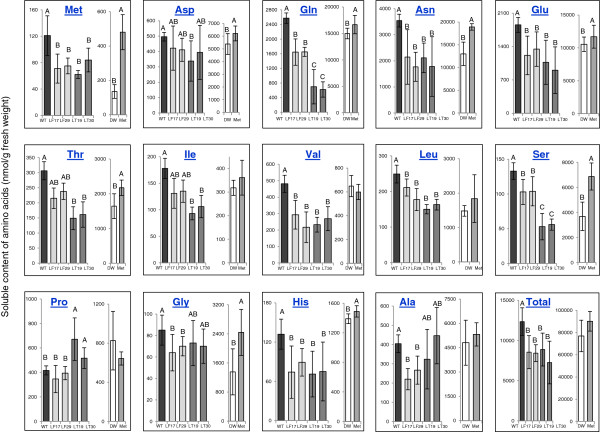
**Soluble amino acid contents in seeds of wild type (WT) and homozygous transgenic tobacco seeds expressing the two forms of *Arabidopsis *CGS: the full-length AtCGS (LF) and the truncated form of AtCGS (LT) whose levels were significantly altered from those found in WT.** Two transgenic events for each line were analyzed (LF17; LF29 and LT19; LT30). The left panel in each box of amino acid described the level of this amino acid in WT and transgenic seeds, while the right panel in each box described the results obtained from the feeding experiments performed on a receptacle of developing capsules of WT fed with 5 mM Met or DDW as a control. The quantities of amino acids were analyzed using GC-MS, and their levels were normalized to norleucine and calculated as nmol per g fresh weight of seeds. The data presented represent the mean ± SD of four samples taken from three different plants for each line. Statistically significant changes (*P* <0.05, using two-way ANOVA) are identified by different letters only when the values were statistically different.

The levels of soluble Met were then measured in seeds of transgenic homozygous lines and WT plants. Unexpectedly, the levels of Met in the two sets of the transgenic seeds did not increase beyond the levels of WT seeds and were even significantly reduced (Figure 3; Additional file 2: Table S1). These results are unlike those reported for tobacco plants overexpressing these two forms of AtCGS, which show significantly higher levels of Met in their leaves [11,12]. In addition, these results also differ from those obtained from soybean and azuki bean seeds expressing the feedback-insensitive forms of AtCGS, which exhibit significantly higher levels of soluble Met (two- to six-fold higher compared to their corresponding WT seeds) [9,10].

The observation that Met does not accumulate in the seeds of the transgenic lines beyond WT levels can be the result of an elevation in catabolic enzymes of Met. An increase in the expression of catabolic enzymes during seed development was reported in tobacco seeds expressing a key enzyme of lysine synthesis [24]. These seeds exhibit significantly higher lysine content compared to WT during their development; however, this triggers the expression of a lysine catabolic enzyme at a later stage of seed development, and eventually the level of lysine does not increase in the dry seeds [24]. We expected that if this phenomenon occurs also in the transgenic seeds expressing the two forms of AtCGS, the level of Met will be increased during seed development compared to WT seeds. To test this, the level of Met was measured during eight stages of seed development. The results show that the levels of soluble Met in the transgenic seeds are similar to those found in WT seeds during all developmental stages (shown for LT seeds, Additional file 1: Figure S3). This suggests that similar processes occur in the WT and the transgenic seeds during seed development, and that the low Met level found in dry transgenic seeds is apparently not related to an elevation in the rate of Met catabolic enzymes. However, in the future, when the sequences of the genes encoded to the two main catabolic enzymes of Met, SAM synthase and Met γ-lyase (Figure 1) from tobacco will be discovered, it will be worthwhile studying if their expression levels and/or activities increase during seed development in the transgenic lines. In addition, the role of the Young cycle and the methyl cycle derived from Met could be studied in these seeds to gain better knowledge of these pathways when the expression level of AtCGS increased in seeds.

### Evidence of enhanced Met synthesis in transgenic seeds during seed development

**Figure 4:**
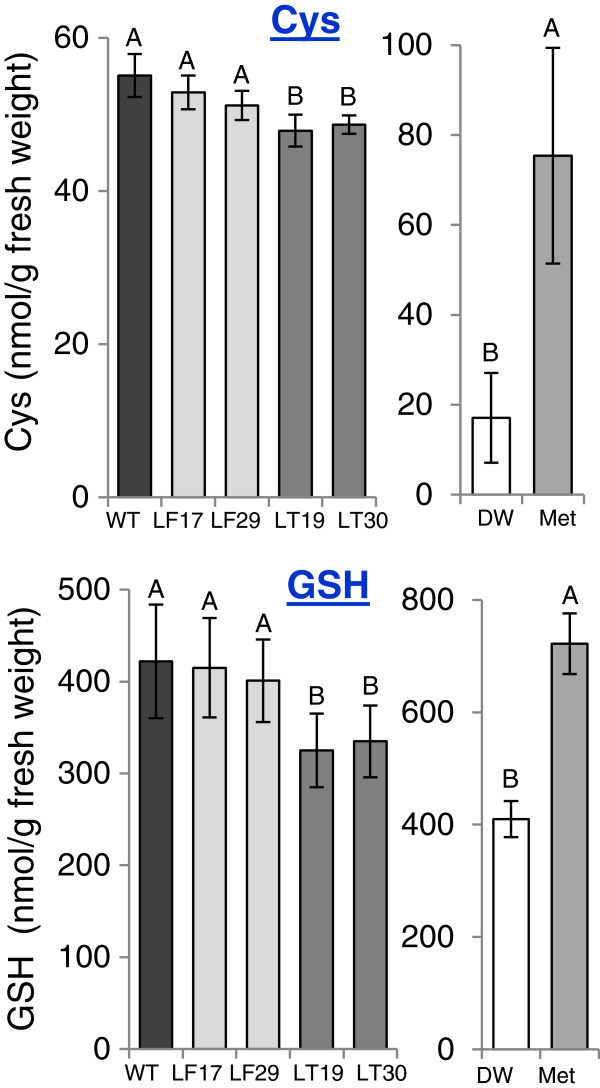
**The level of cysteine (Cys) and glutathione (GSH) in seeds of wild type (WT) and homozygous transgenic tobacco seeds expressing the two forms of *Arabidopsis *CGS: the full-length AtCGS (LF), and the truncated form of AtCGS (LT).** Two transgenic events for each line were analyzed. The left panel in each box described the level of Cys or GSH in WT and transgenic seeds, while the right panel described the results obtained from the feeding experiments performed on a receptacle of developing capsules of WT fed with 5 mM Met or DDW as a control. The quantities of these two metabolites were analyzed using HPLC. The data presented represent the mean ± SD of four samples taken from three different plants for each line. Statistically significant changes (*P* <0.05, using two-way ANOVA) are identified by different letters.

The synthesis of Met is connected with other amino acids such as those belonging to the aspartate family pathway (Figure 1). Since the reduction of soluble Met in the transgenic seeds was unexpected, we have measured the levels of other amino acids to define changes that occur with an increase in expression level of AtCGS. The analysis revealed that in addition to Met, the levels of seven amino acids: glutamine, glutamate, asparagine, serine, valine, leucine and histidine were significantly reduced in both transgenic lines. The levels of threonine, and isoleucine were reduced significantly only in transgenic LT seeds, while alanine and glycine were reduced significantly only in LF seeds. The level of proline increased significantly in LT seeds, compared to WT seeds (Figure 3; Additional file 2: Table S1). From these altered amino acids, eight can be considered as Met-related amino acids (Figure 1). Glutamine and glutamate serve as precursors for aspartate that donates its carbon-amino acid skeleton to Met synthesis. The levels of these amino acids tend to be reduced when the content of Met, as well as levels of lysine and threonine, two additional amino acids that belong to the aspartate family of amino acids (Figure 1), increase [12,24,25]. Threonine competes with Met for their common precursor *O*-phosphohomoserine (Figure 1), and a decrease in the levels of threonine was observed in several plants following an increase in Met levels [1]. The branched amino acids, leucine, isoleucine and valine, are related to Met since the carbon/amino skeleton of these amino acids, is generated by the catabolism of threonine and Met [26-28]. Indeed, their levels increased significantly when high levels of Met were found in the leaves of transgenic tobacco and Arabidopsis plants [12,29]. Serine is the precursor of the carbon/amino skeleton for cysteine synthesis, which is the sulfur donor for Met synthesis. A decrease in serine content was reported when the requirement for cysteine and Met increased [30-32]. The decrease in the level of serine suggests that the level of cysteine was decreased as well. Thus, an HPLC analysis was performed to measure the level of cysteine. The results show that the level of cysteine decreased significantly (p < 0.05) in LT seeds, while in LF seeds, the decrease in cysteine levels was insignificant (Figure 4). Since cysteine is also used as a precursor for the synthesis of glutathione (GSH), and the level of GSH is limited by cysteine (e.g., [33-35]), the level of GSH was also determined. Similarly to cysteine, the level of GSH was insignificantly altered in LF, and reduced significantly in LT compared to WT (Figure 4), a reduction of 22%.

The low levels of the 11 amino acids (including cysteine) in seeds of LT suggest that although we did not detect an increase in the amounts of soluble Met, the flux towards Met synthesis was enhanced in the transgenic seeds, causing a decrease in the content of Met precursors and competitors.

To test this assumption, and to assess whether the 11 amino acids whose levels were reduced in LT seeds, are indeed related to Met synthesis and metabolism, the receptacles of developing capsules of WT plants were fed with Met. It is expected that feeding with Met will reduce the flux towards Met synthesis due to a reduction in the expression level of NtCGS (Figure 2A). If this occurs, we will expect an elevation in the levels of Met precursors (such as aspartate, glutamine and cysteine) or Met competitors (threonine) in the seeds. To test this assumption, receptacles of developing capsules (16–17 days after flowering) were fed with 0.5 ml of 5 mM Met, or with DDW as a control. At this stage of development, the proteins accumulate and amino acids are synthesized in the seeds [16]. Twenty-four hours later, the seeds were collected from these capsules and the levels of free amino acids were analyzed. The values obtained for the soluble amino acids (nmol/g fresh weight) were significantly higher in seeds obtained from the fed capsules compared to those obtained from the dry seeds (comparing the value in each amino acid between the left and right panels in Figure 3). This apparently happens because at that stage of seed development, most of the amino acids are not yet incorporated into the seed-storage proteins.

The results show that the level of Met increased significantly in the seeds from capsules fed with Met (3.5-fold) (Figure 3; Additional file 2: Table S2), indicating that Met can be transported from the receptacle to the seeds. The level of aspartate, glutamate, glutamine, serine (Met precursors), threonine (Met competitor), glycine and histidine were increased significantly in the seeds fed with Met (Figure 3; Additional file 2: Table S2). The levels of homocysteine and homoserine, intermediate metabolites of the Met biosynthesis pathway (Figure 1), also increased significantly (Additional file 2: Table S2), suggesting that under these conditions the activity of Met synthase, the last enzyme of Met synthesis, and homoserine kinase, are regulating the Met synthesis. In addition to these Met-related amino acids, the level of lysine that belongs to the aspartate family together with Met also increased significantly, while the level of tryptophan decreased significantly (Figure 3; Additional file 2: Table S2). The level of cysteine that was determined by HPLC shows that its level increased significantly in those fed with Met compared to those fed with DDW (a 4.4-fold increase). The level of GSH also increased significantly in these seeds at an elevation of 1.7-fold (Figure 4). These results, as well as those shown in Figure 4, suggest that a high expression of T-AtCGS increases the flux of cysteine towards Met synthesis in LT seeds at the expense of cysteine and its tripeptide, GSH.

The alternation in the content of cysteine and GSH in LT and in the Met-fed seeds was unexpected. Thus, to gain more knowledge about these changes, we studied how a high expression of T-AtCGS in LT seeds affects the transcript expression level of two genes in the GSH synthesis. We also determined the transcript level of adenosine-5′-phosphosulfate-reductase, a key enzyme of the cysteine biosynthesis pathway [36,37]. None of the expression levels of these transcripts were altered between WT and LT seeds (Additional file 1: Figure S4). These results suggest that the alternation in GSH content is controlled less by the transcript levels of genes involved in cysteine and GSH biosynthesis pathways, and more by the flux of cysteine towards GSH and/or Met biosynthesis pathways. However, further studies are required to define the role of other factors and enzymes, as well as the role of *O*-acetylserine, the key metabolite that regulates the synthesis of cysteine [36].

Taken together, the data obtained by feeding experiments showed that the reduction of the flux towards Met synthesis caused by Met-feeding, led to an elevation in the levels of Met precursors and its competitors. These results support the assumption that although the level of Met in the transgenic seeds is relatively low, there is an enhanced flux towards Met synthesis during seed development. This is more pronounced in LT than in LF seeds that are feedback-sensitive to Met/SAM [15]. However, additional studies are required to monitor directly the metabolic flux leading to Met synthesis and the effect of T-AtCGS expression on this flux in seeds. The results also indicate that in tobacco seeds there is a connection between high expression levels of T-AtCGS to the levels of 12 amino acids and GSH.

### LT seeds contain higher levels of total Met in their proteins

The results described above suggest that there is a higher flux towards Met synthesis in LT seeds. Since the results described in Additional file 1: Figure S3 imply that Met is converted to other metabolites in LT seeds at the same rate as it occurs in WT seeds, we assume that in LT seeds, Met was used mainly to synthesize storage proteins. Support of this assumption was derived from the observation that the soluble levels of the three branched amino acids that can be produced through Met catabolism [26,27] were not increased and even reduced significantly in LT seeds (Figure 3; Additional file 2: Table S1).

To test the assumption that in LT seeds Met was used to synthesize the seed’s storage proteins, the levels of amino acids were measured after protein hydrolysis in dry seeds of WT, LF and LT. The total Met content in seeds of the two LF lines did not change significantly and were even slightly reduced when compared to WT (Table 1). One possible explanation for this observation is that the Met content in LF seeds cannot be increased further because of the feedback inhibition regulation occurring on AtCGS transcript level by SAM [15]. Moreover, since the level of soluble Met was reduced significantly in LF seeds compared to WT (Figure 3), it also implies that the sensitivity to the feedback inhibition of AtCGS (which is the major form of CGS in transgenic seeds) is higher than the NtCGS, hence reducing the ability of Met to accumulate. To test this assumption further, the expression level of NtCGS was measured in seedlings of WT and transgenic seedlings overexpressing the AtCGS [11]. As expected from the observation that the expression level of NtCGS is sensitive to a high level of Met (Figure 2A, left panel), its level was lower in seedlings overexpressing the F-AtCGS compared to WT (since they have a higher level of Met compared to WT in these plants) (Figure 2A). Next, we also analyzed the effect of a high content of Met on the expression level of AtCGS and NtCGS. The results (Figure 2A, B) showed that while the expression level of NtCGS was reduced by 33%, that of AtCGS was reduced by 52% compared to those fed with DDW. This suggests that AtCGS is more sensitive to feedback inhibition caused by a high level of Met compared to NtCGS. Since the sensitivity of AtCGS to Met is similar in the whole tissues of Arabidopsis [38], we expected that this is also the case for NtCGS. If this is indeed the case, the results described in Figure 2 can explain why the level of Met was reduced in LF transgenic seeds. However, further studies are required to test this assumption and to reveal why the contents of nine additional soluble amino acids (alanine, valine, leucine, glutamine, asparagine, glutamate, serine, glycine and histidine) were significantly decreased in LF transgenic seeds (Figure 3).

**Table 1:** The level of total amino acids in dry wild type (WT) seeds, in seeds expressing F-AtCGS (LF) and in seeds expressing T-AtCGS (LT), after protein hydrolysis

	**WT**	**LF 29**	**LF 17**	**LT 19**	**LT 30**
Alanine	19232 ± 1957	20778 ± 2289	21704 ± 1098	22891 ± 1713	21993 ± 1665
Valine	41269 ± 6044	41332 ± 6002	41269 ± 3322	43454 ± 1418	45885 ± 3045
Serine	18725 ± 1181 B	18090 ± 3097 B	18725 ± 2660 B	28859 ± 130 A	22908 ± 127 A
Leucine	45675 ± 7705	45499 ± 5528	45531 ± 6186	53449 ± 985	55274 ± 1937
Threonine	23410 ± 4732	23187 ± 4212	23410 ± 3357	34249 ± 1026	31440 ± 2247
Isoleucine	30985 ± 4603	31254 ± 1233	31981 ± 1966	32913 ± 599	39781 ± 1098
Proline	11234 ± 1891 B	10954 ± 985 B	11164 ± 1550 B	13196 ± 1439 A	13784 ± 726 A
Glycine	108546 ± 4480	108756 ± 3567	109546 ± 5814	109494 ± 6499	112348 ± 4972
Methionine	4374 ± 315 B	3962 ± 997 B	4199 ± 313 B	6992 ± 459 A	6376 ± 573 A
Aspartate	39625 ± 11058 B	41500 ± 9876 B	40625 ± 7303 B	61360 ± 2182 A	48541 ± 3687 A
Phenylalanine	21500 ± 4772	23058 ± 5562	22003 ± 3568	27960 ± 1025	28166 ± 2235
Glutamate	3058 ± 785 B	2984 ± 689 B	3644 ± 330 B	9217 ± 242 A	5896 ± 564 A
Histidine	2725 ± 666	2769 ± 1072	2863 ± 605	3415 ± 358	3722 ± 231
Lysine	20263 ± 4330	22645 ± 5562	22094 ± 4570	25838 ± 1138	24113 ± 2645
Tyrosine	19841 ± 3273	20476 ± 5797	21195 ± 3165	24230 ± 1115	27420 ± 971
Total	411462 ± 21890 B	417244 ± 18664 B	419953 ± 11284 B	497517 ± 19064 A	487647 ± 13687 A

The levels of amino acids were measured using GC-MS and displayed as nmol/gr dry seeds. Values are representative of two independently grown sets of plants are the mean ± standard deviation of five biological repetitions of 50 mg seeds isolated from three plants per line. Statistically significant changes (p < 0.05, using two-way ANOVA) are identified by letters.

Unlike LF, LT seeds expressing the feedback-insensitive form of AtCGS (T-AtCGS) exhibit significantly higher levels of total Met, which increased 46 to 60%, compared to WT seeds (Table 1). Based on the results obtained from LT, we suggest that the level of soluble Met in tobacco WT seeds limits the Met content that can be incorporated into proteins. Similar assumptions were made in several other studies showing that higher levels of soluble cysteine, lysine, tryptophan or Met in transgenic soybean seeds [10,39-41], and tryptophan in rice seeds [42] lead to an increase in these amino acids in the seeds’ proteins. However, the ability of the higher soluble amino acids to incorporate into proteins appears to differ between different seeds. Significantly higher levels of soluble Met (about 6-fold) led to higher total Met (about 2-fold) in soybean seeds of Zigongdongdou cultivar [10]. Yet, when the soybean cultivars Misuzudaizu and Bert were used, it was reported that although they exhibit significantly higher levels of soluble Met (2-fold), the level of total Met was not altered significantly [9]. Similarly, high levels of soluble threonine (16-fold) in tobacco seeds [24] and high levels of cysteine in lupine seeds (26-fold) [43] do not alter the total threonine or cysteine in the transgenic seeds [43,44].

### LT seeds have higher levels of total amino acids and proteins

In addition to the significantly higher levels of total Met in LT seeds, the total levels of aspartate, glutamate and two stress-associated amino acids, proline and serine also increased significantly in LT seeds (Table 1). Furthermore, the levels of most other amino acids in LT seeds were increased, although insignificantly. As a result, the total amino acids content increased significantly in LT seeds by 18-21% (Table 1). This elevation can explain why the levels of total soluble amino acids decrease significantly (Figure 3; Additional file 2: Table S1). A similar trend of higher total amino acids was also found in two transgenic soybean seeds expressing the feedback-insensitive form of AtCGS, in which the level of total amino acids increased by 55% and 76% [10]. However, this is not a general phenomenon in other amino acids, since transgenic soybean seeds having higher levels of total tryptophan and Arabidopsis seeds having a higher level of lysine did not show an increase in total amino acids levels [40,45]. This suggests that Met level is a limiting factor of protein synthesis in tobacco seeds, and that when Met synthesis is enhanced, most of the other soluble amino acids are able to incorporate into proteins. However, further studies are required to define the mechanism behind this phenotype and to determine if an elevation in the content of other yet unstudied amino acids can also lead to the same phenomenon.

The increase in the amount of protein-incorporated amino acids suggests that LT seeds have higher protein content. To test this possibility, the amounts of water-soluble proteins in the four samples of seeds were measured from groups of 10 mg seeds from each line. The results revealed that LT seeds have a higher content of proteins (2.22 ± 0.36 μg/μl in LT compared to 1.74 ± 0.39 μg/μl in WT, an increase of 27%, which is statistically insignificantly).

### The effect of higher expression levels of T-AtCGS on the seeds’ primary metabolites

The expression of T-AtCGS leads to an elevated level of total Met (Table 1) and altered levels of 12 soluble amino acids (Additional file 2: Table S1). In order to assess whether the higher flux towards Met synthesis is associated with additional changes in the levels of primary metabolites, we performed a metabolic profiling analysis using established gas chromatography–mass spectrometry (GC-MS) [46-48]. Despite the significant changes in seed metabolism that occur during seed development, little is known regarding changes to the metabolic profile of the seed during development when heterologous genes are expressed [17,18]. Therefore, we were interested in studying whether a perturbation in Met metabolism influences individual metabolites within the seeds.

The GC-MS analyses revealed 102 peaks having a significantly higher signal-to-noise value. In order to identify the chemical nature of as many peaks as possible, standards were used. In addition, the spectra of all the peaks were compared with commercially available electron mass spectrum libraries, NIST and WILEY. Fifty-two annotated specific compounds were detected in WT and transgenic seeds (Additional file 3: Table S4), several of which appeared at more than one retention time.

**Figure 5:**
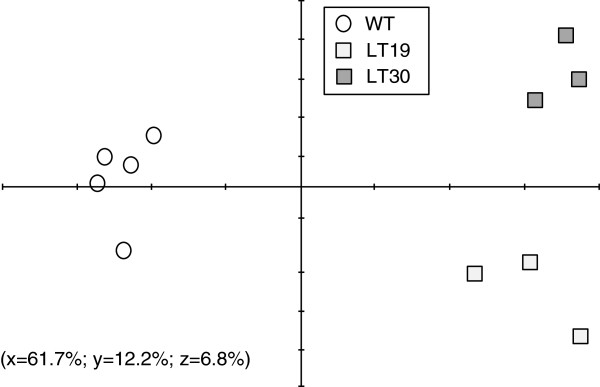
**Principal component analysis (PCA) of 102 peaks that represent different metabolites measured in wild-type (WT) and LT transgenic seeds expressing the T-AtCGS gene.** PCA is presented as a combination of the first three dimensions, which together comprise 80.7% of the metabolite variance. Each data point represents an independent sample. The data analysis was performed using TMEV software. Component 1 explained 61.7% of the variance, component 2 12.2%, and component 3 6.8%. The combined percentages of the variance are given in brackets.

When analyzing the trends of the changes to metabolites concentration in seeds by principal component analysis (PCA), the WT and LT genotypes exhibited clearly distinguishable differences, implying a relatively strong effect of the genetic manipulation on the primary metabolism of LT seeds (Figure 5). These results are dissimilar to those reported in Arabidopsis seeds having higher levels of lysine, which show little differences from WT [17]. The PCA analysis allowed us to define the metabolites and peaks that contributed significantly to the variance between WT and LT genotypes. Thus, these metabolites and peaks were also assessed by a two-way ANOVA.

**Figure 6:**
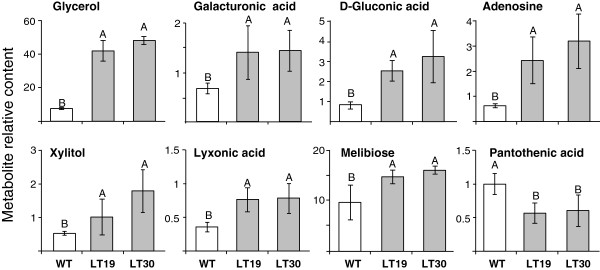
**Relative contents of the eight annotated metabolites whose levels were altered significantly (according to two-way ANOVA) in dry seeds of the LT genotype (LT 19 and LT 30), compared with wild-type (WT) seeds.** Values represent the response of each metabolite expressed as peak area normalized to the internal standard norleucine and ribitol. Values are representative of two independently grown sets of plants and are presented as the mean ± SD of five biological repetitions of 50 mg isolated seeds from three plants from each line. Statistically significant changes (*P* <0.05, using two-way ANOVA) are identified by different letters.

Among the 52 annotated metabolites, the levels of seven metabolites were significantly higher in LT compared to WT seeds: glycerol, gluconic acid, galacturonic acid, xylitol, lyxonic acid, melibiose and adenosine, while that of pantothenic acid was reduced significantly (Figure 6). In addition, four peaks (17, 31, 72, 73; Additional file 3: Table S4), decreased significantly in LT seeds, while two others (90, 93) increased significantly. None of the annotated metabolites are known according to the literature to be related to Met. Most of the compounds whose levels increased in LT are sugars or their oxidized derivatives. Gluconic acid is the oxidized form of glucose, and galacturonic acid, which is the main component of pectin, is an oxidized form of galactose. Xylitol and lyxonic acid (also called xylonite) are produced from xylose by hydrogenation or mild oxidation, respectively. The higher levels of the oxidized metabolites, as well as higher levels of soluble proline (Figure 3; Additional file 2: Table S1), and total level of serine, which are stress induced amino acids [49,50], imply that oxidative stress occurs during seed development of LT seeds.

In addition to these sugars and their derivatives, the level of adenosine (a purine nucleoside) also increased. Adenosine is known to elicit numerous physiological responses in plants [51,52], and is required for the synthesis of the main Met metabolite, SAM. Purines require folic acid for their synthesis, which is also necessary for the synthesis of methyl-tetrahydrofolate, the substrate for Met synthase, the last enzyme of Met synthesis. However, this is a putative link and further studies are required to reveal this link between Met to adenosine.

While the levels of these metabolites were increased, the content of pantothenic acid (vitamin B_5_) was reduced in LT seeds. Pantothenic acid is the precursor of coenzyme A and is synthesized from aspartate [53], the carbon/amino donor of Met. Thus, a decrease in soluble aspartate levels in LT seeds (Additional file 2: Table S1) might leave less aspartate available for the synthesis of pantothenic acid. Another putative link between Met and pantothenic acid is that they both require folate-dependent enzymes for their synthesis [54]. Thus, when the level of Met increases (and also adenosine), less folate is available for the synthesis of pantothenic acid. However, again further studies are required to clarify these relationships.

To better understand the relationships between these metabolites and the Met metabolism, the WT seeds whose developing capsules were fed with Met were analyzed using the GC-MS scan method. This allowed us to detect the metabolites whose levels were altered 24 h after feeding. The results show that the levels of xylitol and melibiose increased significantly in seeds fed with Met (32% and 27%, respectively). The level of adenosine increased by 15% but was statistically insignificant. The levels of the other compounds did not change significantly compared to seeds fed with DDW. Most probably, the short period of exposure to high Met level is not enough to alter the contents of these metabolites in a significant way. However, this analysis strengthens the assumption that a link exists between xylitol and melibiose to high expression levels of AtCGS in tobacco seeds and to higher level of Met.

### The level of lipids and total reducing sugars in LT seeds

The significant increase in glycerol content in LT seeds may affect lipids content, since glycerol is a precursor for triacylglycerol and may play a role in regulating oil content in seeds [55,56]. In addition, the higher level of protein in these seeds may also affect the level of lipids and starch, as was previously reported for soybean seeds [57]. We have used the Soxhlet method to measure oil content. This analysis revealed that the oil content of LT seeds is slightly higher than that of WT, but this increase was statistically insignificant (40.71% ± 2.46 and 36.75% ± 2.29, respectively).

Since the level of total amino acids in proteins had increased in LT seeds and the level of oil had decreased slightly, we also measured the level of total reducing sugars following carbohydrate hydrolysis, which represents starch content. The content of reducing sugars was slightly but significantly lower in LT seeds than in WT seeds (10.65 ± 0.31 and 11.84 ± 0.13 mg per 1 g dry seeds, respectively). This suggests that the higher protein content was at the expense of starch accumulation. This decrease in total reducing sugars may also be associated with the observation that besides amino acids, most of the metabolites whose levels were altered significantly in LT seeds were sugars and their derivatives (Figure 6). A putative link is that the carbon skeleton of glucoses was modified to other sugars, leaving less glucose available to be incorporated into starch.

### The germination rate of LT seeds is slightly reduced

The extent of the changes in the contents of cellular constituents and key metabolites, which include 20 annotated primary metabolites (including amino acids), total proteins, lipids and reducing sugars in LT seeds, may affect the seeds’ phenotype and their germination rate. Thus, we compared the morphological phenotypes of WT and LT seeds and found no significant differences. The average weight of 100 LT seeds decreased compared to WT seeds (87.5 ± 0.64 mg in LT compared to 93.6 ± 1.19 mg in WT seeds), but this decrease was statistically insignificant. LT seeds had a lower germination rate compared to WT five days after imbibition (Additional file 1: Figure S5), however, four days later, the growth of WT and LT seedlings was indistinguishable. A decrease in germination rate of soybean and Arabidopsis seeds having increased levels of lysine was reported [41,45], however, that of soybean with an increased level of threonine [58] or Met [10] was not affected. Hence, different seeds respond in different ways to a high flux or to high level of soluble amino acids. In general, the results of the current study suggest that the germination rate of tobacco seeds is relatively tolerant to the perturbations that occur when the Met synthesis increases, causing significant changes to the profile of 20 annotated metabolites, as well as in the levels of total reducing sugars and proteins.

## Conclusions

Based on the results described above, we propose the following explanation for the changes that occur in tobacco seeds following the expression of AtCGS: a high expression level of the Met/SAM feedback-insensitive form of AtCGS in LT seeds, unlike the feedback-sensitive form of AtCGS in LF seeds, leads to a significant increase in flux towards Met synthesis. This leads to a significant increase of Met synthesis, that causes a reduction to the contents of amino acids that serve as precursors for Met. The increase of Met synthesis enhanced the protein synthesis rate, which can utilize other soluble amino acids for incorporation into storage proteins during seed maturation. As a result, the amount of total amino acids increased (Table 1), as well as the amount of total water soluble proteins (by about 27%). This suggests that CGS plays a role in Met synthesis in tobacco seeds as was found in legumes seeds [9,10,59]. However, further studies are required to assess whether Met can also be synthesized from SMM at the latter stages of seed development, as was suggested for *Medicago truncatula*[60].

In addition to our goal to assess the role of AtCGS in Met synthesis in seeds, we also aimed at deciphering how this manipulation cross-interacts with other primary metabolites in the seeds. The results show that the levels of 20 annotated primary metabolites (including amino acids) were altered in LT in comparison to WT seeds. These include 12 metabolites whose levels decreased and 8 metabolites whose levels increased. The results also indicate that unlike lysine metabolism, Met metabolism has a relatively large impact on primary metabolites, since high lysine content affected only four metabolites connected to the TCA cycle [17]. In general, the levels of most free amino acids were reduced in LT, while the levels of sugars and their derivates were elevated. The increase in oxidized sugars and the higher levels of total proline and serine, two amino acids that their level increase during oxidative stress [49,50], suggest that LT seeds suffer from oxidative stress. The oxidative stress might be related to the low content of GSH, a major antioxidant compound [61], in LT seeds. However, further studies are required to clarify the relations between high level of T-AtCGS, low GSH and oxidative stress, during seed development.

This study provides information on the metabolic consequences of enhancing the expression level of CGS is in seeds. In addition, since the levels of total soluble proteins increase in LT seeds by 27%, the total lipid content was reduced by about 4% (although insignificantly), and the total levels of reducing sugars, which represent starch content, was reduced significantly by 4.6%, we also define that the high expression level of T-AtCGS affects the seed’s nutritional quality.

## Methods

### Generation of transgenic plants expressing different forms of AtCGS in a seed-specific manner

The binary constructs were prepared as described in Song et al. [10] and in Additional file 1: Figure S1. Tobacco plants (*Nicotiana tabacum* cv Samsun NN) were transformed with these constructs as previously described [11]. The plants were grown in a growth chamber under 16/8 hours light/dark cycle and temperature of 23-27°C. Heterozygous mature T_0_ seeds from 30 kanamycin-resistant plants from each transgenic line were screened for protein expression level by immunoblot analysis [11]. Two plants having the highest expression levels from each set were chosen for further study. These plants were self-pollinated to obtain homozygous plants.

### Extraction, derivatization and analysis of seed amino acids

Seeds from at least 15 capsules collected from each of the transgenic and WT lines were pooled. Free amino acids were extracted from 20 mg of dry seeds. For total amino acid determination including protein-bound amino acids, 10 mg of dry seeds were taken as described [30]. For acid hydrolysis, a Carousel 12 plus reaction station (Radleys, UK) was used. The amino acids were detected using the single ion method (SIM) of GC-MS, as previously described [30]. For cysteine and GSH determination, Dry seeds (50 mg) were ground with a mortar and pestle and then extracted and analyzed by HPLC, as previously described [33].

### GC-MS instrumentation and data analysis of primary metabolites using GC-MS

For primary metabolites analysis, samples were prepared as described for the free amino acids [30] and 7 μl of a retention time standard mixture (0.2 mg/ml n-dodecane, n-pentadecane, n-nonadecane, n-docosane and n-octacosane, in pyridine); in addition, 4.6 μl of a retention time standard mixture of norleucine and ribitol (2 mg/ml) were added prior to trimethylsilylation.

Samples were run on a GC-MS system (Agilent 7890A series GC system coupled with Agilent 5975c Mass Selective Detector), and a Gerstel® multipurpose sampler (MPS2) was installed on this system. The analysis method was adapted to our system based on Roessner et al. (2001) [46]. Helium was used as the carrier gas at a flow rate of 1 mL per min. 1 μL was injected in the splitless mode, the inlet temperature was 230°C and the pressure was 8.805 psi. The GC was performed on a 30 m RXI-5-Sil MS Capillary Column with a 0.25 mm inner diameter and 0.25 μm film thickness (Restek, USA). The analysis was performed under the following temperature program: 5 min of isothermal heating at 70°C followed by a 5°C min^-1^ oven temperature gradient to 310°C, and a final 3 min of heating at 310°C. The system was then temperature equilibrated for 6 min at 70°C before the injection of the next sample. Mass spectra were recorded at 2 scan sec^-1^ with a scanning range of 50 to 600 m/z.

Standards substances (most of them kindly provided by Asaph Aharoni, Weizmann Institute) were dissolved in 50% methanol (1 mg/ml). A 5 μl volume of standard solution was dried under vacuum and derivatized with 40 μl of 20 mg/ml methoxyamine hydrochloride in pyridine and 100 μl N-methyl-N-(trimethylsilyl) trifluoroacetamide (MSTFA). The data collected were obtained using Agilent GC/MSD Productivity ChemStation software. All peaks above the baseline threshold were quantified and grouped according to retention time, with areas normalized to norleucine and ribitol. Substances were identified by comparison with standards, and were also compared with commercially available electron mass spectrum libraries NIST and WILEY.

### Application of Met to developing capsules

Half ml of 5 mM Met in sterile DDW was injected into the receptacle of developing capsules of WT 16–17 days after flowering. At this stage, seed color turned from white to light brown. After 24 hr, the capsules were harvested and kept on ice. Seeds were separated from the capsules, dried using a lyophilizer and stored at −70°C until used. Capsules injected with DDW were used as control.

### RNA extraction from tobacco seedlings and expression analysis

For RNA extraction, plants were grown in Nitsch agar plates [Nitsch medium (DUCHEFA), pH adjusted to 5.8 with KOH 0.9% (w/v) containing 2% sucrose] at light/dark cycle (16 h/8 h). Twenty-one-day-old tobacco seedlings were fed with 5 mM Met or with DDW as a control. After 6 h, plants were frozen in liquid nitrogen as a pool of 30 seedlings in five biological repeats and kept until use at −80°C. For qRT-PCR, the RNA was extracted and cDNA was synthesized as previously described [10]. Primes used in the qRT-PCR analysis are summarized in Additional file 2: Table S3. To normalize the variance among samples, the PP2A transcript level was used as endogenous control [62]. The values presented are the mean of three biological replicates.

### Determination of germination rates of transgenic LT and WT seeds

Seeds were placed overnight in water at 12°C with gentle shaking and then transferred to Petri dishes with Watmann paper wetted with water. The plates were placed at 25°C in a standard plant growth chamber under a 16/8-h light/dark regime for 10 days.

### Total protein, lipid and reduced sugars determination in seeds

For total protein determination, 10 mg seeds were ground in 120 μl of 25 mM Na phosphate buffer pH = 7.8 with a protease inhibitor cocktail (Sigma, P9599). After two centrifugation cycles (14,000 rpm 4°C for 20 min), total protein was determined using a Bradford reagent (Bio-Rad Hercules, Calif). Bovine serum albumin was used as a standard.

The Soxhlet method was used for lipid determination as previously described [10]. Reducing sugars were measured after carbohydrates and starch hydrolysis calorimetrically using the Sumner method [63]. 150 mg of seeds were dried and ground as described above. The seeds were extracted in 80 ml of HCl 1 N at 70°C for 2 h. After cooling, the pH was corrected to 7.5 and the volume corrected to 100 ml with water. 0.1 and 0.2 ml of the samples were then mixed with 1.9 and 1.8 ml water, respectively, and 2 ml of Sumner reagent was added. The reducing sugars were detected at 550 nm.

### Statistical and network analyses

The data obtained from this study were analyzed statistically using JMP 8 software. In this software, we used ANOVA and the Student’s t-test programs, as described in the text. A P value of <0.05 was considered statistically significant.

For the statistical analysis required for the metabolic profile analysis, PCA and two-way ANOVA were performed on the datasets obtained from metabolite profiling with the software package TMEV [64] using the default weighted covariance-estimation function. Prior to the analysis, data were log transformed and normalized to the median of the entire sample set for each metabolite. To test statistical significance between specific time points, t-tests were performed using the algorithm incorporated into Microsoft Excel. We set the correlation significance threshold fixed at the level of the Spearman correlation coefficient.

## Abbreviations

Met: Methionine; SAM: *S*-adenosyl methionine; SMM: *S*-methyl methionine; CGS: Cystathionine γ-synthase; AtCGS: Arabidopsis CGS; WT: Wild type; GSH: Glutathione; qRT-PCR: Quantitative real time PCR; GC-MS: Gas chromatography–mass spectrometry; DDW: Double distilled water.

## Competing interests

The authors declare that they have no competing interests.

## Authors’ contributions

IM designed and performed the construction of the binary construct, the expression level analysis of the transgenic seeds, together with IG they analysed the amino acids content and performed the feeding experiments; YH carried out the molecular genetic studies in tobacco plants, and performed the statistical analysis; RA designed and coordinated the study and wrote the manuscript. All authors read and approved the final manuscript.

## Authors’ information

RA is Associated Professor at Tel Hai Collage and Migal Research Institute, Israel. In the last years she mainly studied the metabolism of Met in plants. Dr. YH, and IM are part of her research team. IG is a MSc. student.

## Supplementary Material

Additional file 1: Figure S1 (a) Schematic presentation of Arabidopsis cystathionine γ-synthase (AtCGS) protein, and the constructs used in this study. **Figure S2**: Samples of T_1_ screening of transgenic seeds LF and LT seeds by immunoblot analysis. **Figure S3**: The level of soluble Met in WT and transgenic LT seeds during seed development. **Figure S4**: Quantitative real-time PCR analyses of representative genes in the cysteine and GSH biosynthesis in LT and WT seeds. **Figure S5**: The germination rate of LT seeds. Click here for file

Additional file 2: Table S1 Soluble amino acid contents in seeds of WT and homozygous transgenic tobacco seeds of LF and LT. **Table S2**: Soluble amino acid contents in WT tobacco seeds after application of double distilled water (DDW) or 5 mM methionine in DDW, to the receptacle of developing capsules. **Table S3**: List of primers used in this study. Click here for file

Additional file 3: Table S4. The entire data set of the relative contents of metabolites in WT, LF and LT seeds. Click here for file
