# Supplementary material for: Tobacco seeds expressing feedback-insensitive cystathionine gamma-synthase exhibit elevated content of methionine and altered primary metabolic profile
Source: BMC Plant Biol. 2013 Dec 7;13:206. doi: 10.1186/1471-2229-13-206 (PMC3878949; doi:10.1186/1471-2229-13-206)
Supplement: Additional file 1: Figure S1 — (a) Schematic presentation of Arabidopsis cystathionine γ-synthase (AtCGS) protein, and the constructs used in this study. Figure S2: Samples of T1 screening of transgenic seeds LF and LT seeds by immunoblot analysis. Figure S3: The level of soluble Met in WT and transgenic LT seeds during seed development. Figure S4: Quantitative real-time PCR analyses of representative genes in the cysteine and GSH biosynthesis in LT and WT seeds. Figure S5: The germination rate of LT seeds. [file 1471-2229-13-206-S1.docx]

**Additional file 1: Figure S1**


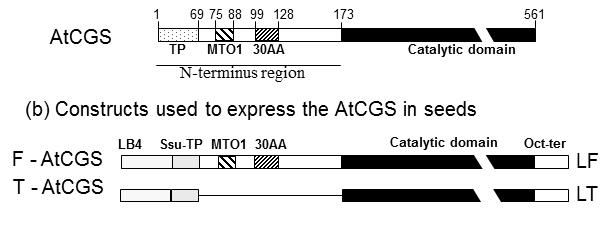


**Figure S1:** (a) Schematic presentation of the Arabidopsis cystathionine γ-synthase (AtCGS) protein. The amino acids are numbered from the first Met of the transit peptide. Key: TP, the chloroplast-targeting transit peptide that directs the protein into the chloroplast and is then removed; N-terminus, the N-terminus region of AtCGS (following the removal of the transit peptide) that shares no homology to CGSs of bacteria; MTO1, the MTO1 domain responsible for the AtCGS transcript degradation in response to a high level of *S*-adenosylMet (SAM); 30 AA, the 30 amino-acid domain, the omission of which leads to the Met-insensitive form of AtCGS catalytic region, the part of AtCGS sharing a homology to bacteria CGSs and harboring the catalytic site; (b) The constructs used to express the full-length CGS (F-AtCGS) or truncated form of CGS lacking the N-terminus region (T-AtCGS) under the control of the Legumin B4 promoter and the octapine synthase terminator (Oct-ter) producing the LF and LT transgenic plants.

**Additional file 1: Figure S2**

**
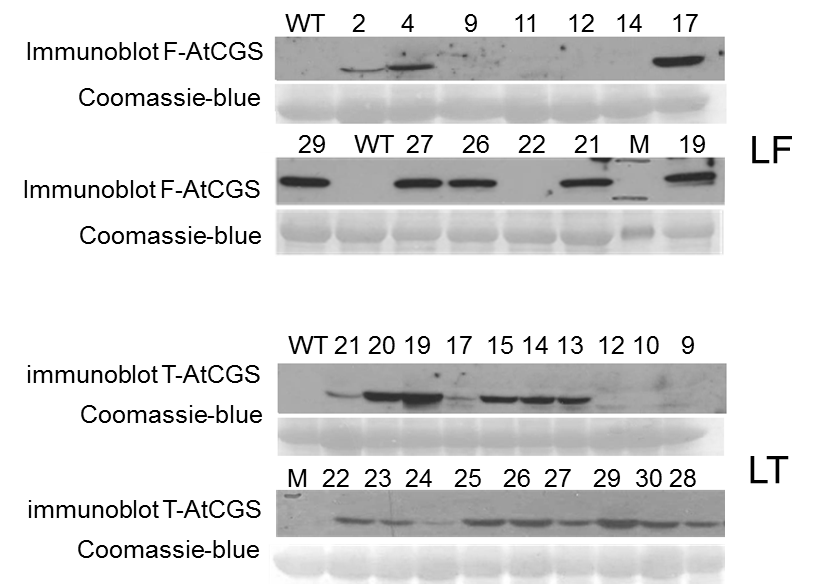
**

**Figure S2:** Samples of T_1_ screening of transgenic seeds expressing the full-length (LF) or truncated (LT) forms of AtCGS by immunoblot analysis using antibodies against the AtCGS. Coomassie-blue staining of a band that is one of the major proteins in the seeds was used for equal loading. M, marker size; WT, wild type seeds.

**Additional file 1: Figure S3**


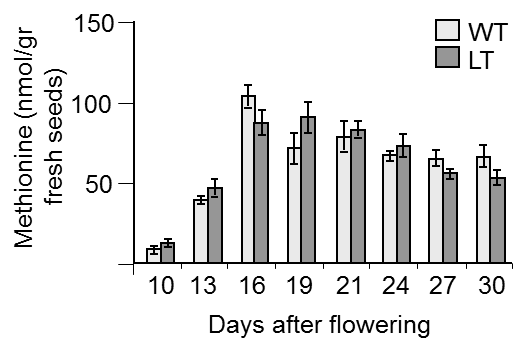


**Figure S3:** The level of soluble Met in wild-type (WT) and transgenic seeds expressing the T-AtCGS (LT) during seed development.

**Additional file 1: Figure S4**

Relative expression

Ntγ-ECS NtGSH-S NtAPR

WT

T-AtCGS

**Figure S4:** Relative expression of two genes in the GSH biosynthesis pathway: γ-EC synthetase (Ntγ-ECS) and Glutathione synthetase (NtGSH-S), and in cysteine synthesis: Adenosine 5’-phosphosulphate reductase (NtAPR), as detected by quantitative real-time PCR analysis in wild type (WT) and transgenic seeds expressing the T-AtCGS (LT). The values presented are the mean ± standard deviation of three biological replicates, each with three technical replicates.

**Additional file 1: Figure S5**


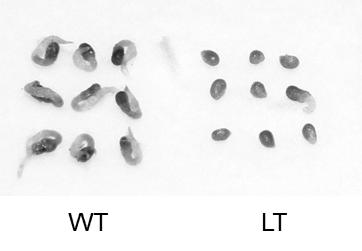


**Figure S5:** The germination rate of five-day-old wild-type (WT) and transgenic tobacco seedlings expressing T-AtCGS (LT30).
