# Supplementary material for: Tobacco seeds expressing feedback-insensitive cystathionine gamma-synthase exhibit elevated content of methionine and altered primary metabolic profile
Source: BMC Plant Biol. 2013 Dec 7;13:206. doi: 10.1186/1471-2229-13-206 (PMC3878949; doi:10.1186/1471-2229-13-206)
Supplement: Additional file 2: Table S1 — Soluble amino acid contents in seeds of WT and homozygous transgenic tobacco seeds of LF and LT. Table S2: Soluble amino acid contents in WT tobacco seeds after application of double distilled water (DDW) or 5 mM methionine in DDW, to the receptacle of developing capsules. Table S3: List of primers used in this study. [file 1471-2229-13-206-S2.docx]

**Additional file 2: Table S1**: Soluble amino acid contents in seeds of wild type (WT) and homozygous transgenic tobacco plants seeds expressing the two forms of *Arabidopsis* CGS: the full-length AtCGS (LF), and the truncated form of AtCGS (LT). Two transgenic events for each line were analyzed. Values (nmol/gr dry seeds) are representing the mean ± standard deviation of four biological samples of 50 mg seeds isolated from three repetitions plants. Statistically significant changes (p<0.05, using two-way ANOVA) are identified by letters only when the values were found to be statistically different.

|  | WT | LF 17 | LF 29 | LT 19 | LT 30 |
| --- | --- | --- | --- | --- | --- |
| Alanine | 404 ± 46 A | 220 ± 55 B | 267 ± 73 B | 324 ± 155 AB | 446 ± 146 AB |
| Valine | 481 ± 52 A | 293 ± 87 B | 217 ± 93 B | 233 ± 45 B | 269 ± 73 B |
| Serine | 133 ± 12 A | 103 ± 18 B | 104 ± 26 B | 53 ± 19 C | 56 ± 8 C |
| Leucine | 249 ± 25 A | 211 ± 24 B | 177 ± 31 B | 150 ± 13 B | 163 ± 15 B |
| Threonine | 306 ± 30 A | 215 ± 34 AB | 238 ± 27 AB | 149 ± 38 B | 161 ± 42 B |
| Isoleucine | 178 ± 19 A | 131 ± 28 AB | 135 ± 21 AB | 93 ± 12 B | 106 ± 21 B |
| Proline | 416 ± 37 B | 346 ± 111 B | 394 ± 54 B | 671 ± 174 A | 516 ± 85 A |
| Glycine | 85 ± 44 A | 64 ± 17 B | 70 ± 9 B | 73 ± 21 AB | 80 ± 68 AB |
| Methionine | 121 ± 30 A | 71 ± 22 B | 75 ± 12 B | 62 ± 6 B | 84 ± 18 B |
| Aspartate | 496 ± 28 A | 422 ± 144 AB | 411 ± 75 AB | 338 ± 131 B | 394 ± 176 B |
| Phenylalanine | 151 ± 17 | 134 ± 9 | 142 ± 19 | 114 ± 18 | 122 ± 11 |
| Glutamate | 1854 ± 156 A | 1214 ± 405 B | 1345 ± 362 B | 1069 ± 463 B | 902 ± 489 B |
| Asparagine | 3543 ± 246 A | 2149 ± 1045 B | 1770 ± 557 B | 2116 ± 567 B | 1783 ± 1142 B |
| Glutamine | 2575 ± 142 A | 1644 ± 361 B | 1652 ± 123 B | 698 ± 470 C | 622 ± 209 C |
| Histidine | 132 ± 23 A | 74 ± 40 B | 89 ± 21 B | 71 ± 35 B | 75 ± 45 B |
| Lysine | 16 ± 4 AB | 10 ± 4 B | 11 ± 3 B | 7 ± 3 B | 10 ± 5 B |
| Thyrosine | 235 ± 30 AB | 163 ± 35 B | 188 ± 29 B | 252 ± 100 AB | 209 ± 131 AB |
| Tryptophan | 917 ± 47 B | 1159 ± 86 AB | 1062 ± 117 AB | 1330 ± 62 AB | 1120 ± 165 AB |
| Total | 12349 ± 1715A | 8547 ± 2048 B | 8427 ± 1048 B | 8873 ± 2048 B | 7259 ± 2653 B |

**Additional file 2: Table S2:** Soluble amino acid contents in wild type tobacco seeds after application of double distilled water (DDW) or 5 mM methionine in DDW, to the receptacle of developing capsules. Values (nmol/g fresh weight) are of five biological repetitions, each repeat containing 10 mg seeds pool isolated from ten capsules. Statistically significant changes (p<0.05, using two-way ANOVA) are identified by letters only when the values were found to be statistically different.

|  | DDW (control) | 5mM Met |
| --- | --- | --- |
| Alanine | 4808 ± 1389 | 5320 ± 717 |
| Valine | 649 ± 89 | 599 ± 64 |
| Serine | 3691 ± 1137 B | 6869± 1081 A |
| Leucine | 1482 ± 153 | 1840 ± 693 |
| Threonine | 1642 ± 357 B | 2172 ± 224 A |
| Isoleucine | 318 ± 32 | 361 ± 74 |
| Proline | 826 ± 299 | 644 ± 67 |
| Glycine | 1354 ± 633 B | 2438 ± 633 A |
| Homoserine | 38 ± 18 B | 92 ± 14 A |
| Methionine | 135 ± 40 B | 481 ± 102 A |
| Aspartate | 5386 ± 818 B | 6215 ± 589 A |
| Phenylalanine | 211 ± 49 | 213 ± 41 |
| Glutamate | 10551 ± 827 B | 11700 ± 508 A |
| Homocysteine | 435 ± 91 B | 1087 ± 353 A |
| Asparagine | 13002 ± 2587 B | 18918 ± 702 A |
| Glutamine | 14979 ± 3008 B | 16659 ± 1743 A |
| Histidine | 1390 ± 66 B | 1489 ± 77 A |
| Lysine | 6229 ± 1322 B | 9631 ± 1512 A |
| Thyrosine | 439 ± 36 | 412 ± 32 |
| Tryptophan | 127 ± 47 A | 72 ± 13 B |
| Total | 76899 ±14205 | 90130 ± 8895 |

**Additional file 2: Table S3:** The list of primers used for the qRT-PCR analysis in this study.

|  | **Gene** | **Accession Number** | **Orientation** | **Sequence** |
| --- | --- | --- | --- | --- |
| 1 | NtCGS | AF097180 | forward | 5' CGCTAT TCATGCCG GTG AAAGATT 3' |
| 2 |  |  | reveres | 5' AAAACTTGCGCGTCTTTTCTCCTT 3' |
| 3 | AtCGS | U43709 | forward | 5' GGTTTGCATTGATGGCACCT 3' |
| 4 |  |  | reveres | 5' GCGTTTGGGTTAAGTGTT CCTC 3' |
| 5 | NtAPR | AY648056 | forward | 5' CATGTTCCCTGACGCTGTTGA 3' |
| 6 |  |  | reveres | 5' TCACACGGCAGCACTCTTGGTG 3' |
| 7 | Ntg-ECS | DQ444219 | forward | 5' TAATGCCGAAGGGGAGATACG 3' |
| 8 |  |  | reveres | 5' GAGCTGTAGCAATAGGCTGCAAGGC 3' |
| 9 | putative 574-bp NtGSH-S | EB437755 | forward | 5' ACAGTCGGGTTACTTGATGCGGACA 3' |
| 10 |  |  | reveres | 5' CTCCGCATTCAACCCCAGTA 3' |
| **11** | NtPP2A | X97913 | forward | 5' TGAAGGACGAGTTT CCTGATGTGC 3' |
| **12** |  |  | reveres | 5' CGCCAATGCCTGTCCTCTGCCA 3' |
